# Post-neurosurgical meningitis; gram negative bacilli vs. gram positive cocci

**DOI:** 10.22088/cjim.13.3.469

**Published:** 2022

**Authors:** Mehdi Zeinalizadeh, Roya Yazdani, Mohammad Mehdi Feizabadi, Maryam Shadkam, Arash Seifi, Seyed Ali Dehghan Manshadi, Alireza Abdollahi, Mohammadreza Salehi

**Affiliations:** 1 Department of Neurosurgery, Brain and Spinal Cord Injury Research Center, Imam Khomeini Hospital, Tehran University of Medical Sciences, Tehran, Iran; 2 Department of Neurology, School of Medicine, Iran University of Medical Sciences, Tehran, Iran; 3 Department of Microbiology, School of Medicine, Tehran University of Medical Sciences, Tehran, Iran; 4 Department of Microbiology, Imam Khomeini Hospital, Tehran University of Medical Sciences, Tehran, Iran; 5 Department of Infectious Diseases, Imam Khomeini Hospital, Tehran University of Medical Sciences, Tehran, Iran; 6 Department of Pathology, Imam Khomeini Hospital, Tehran University of Medical Sciences, Tehran, Iran

**Keywords:** Nosocomial infections, Neurosurgery, Meningitis, Gram negative bacilli

## Abstract

**Background:**

Post-neurosurgical meningitis is a significant cause of mortality and morbidity. In this study we aimed to compare the differences of clinical, laboratory features and outcomes between the post-neurosurgical meningitis caused by gram-negative bacilli (GNB) and gram-positive cocci (GPC).

**Methods:**

Cases of post-neurosurgical meningitis (with positive CSF culture) were included. After classifying patients as GNB and GPC groups, clinical and paraclinical data were compared.

**Results:**

Out of 2667 neurosurgical patients, CSF culture was positive in 45 patients. 25 (54.3%) were GNB, 19 (41.3%) GPC. The most common microorganisms were *Klebsiella pneumoniae* (n=14, 31.1%),* Coagulase negative staphylococcus* (n=8, 17.8%), *Staphylococcus aureus* (n=6, 13.3%), *Acinetobacter baumannii* (n=4, 8.9%), *Pseudomonas aeruginosa* (n=2, 4.4%), and *Escherichia coli* (n=2, 4.4%). There were no correlation between CSF Leakage, Surgical site appearance, presence of drain, Age and GCS between two groups (*P*=0.11, *P*=0.28, *P*=0.06,* P*=0.86, *P*=0.11 respectively). The only different laboratory indexes were ESR (86.8 mm/h vs. 59.5 mm/h, *P*=0.01) and PCT (13.1 ng/ml vs. 0.8 ng/ml, *P*=0.02) which were higher in GNB cases. 20% (n=5) of patients with GNB meningitis received preoperative corticosteroid, while none of GPC cases received (*P*=0.03). The median length of hospitalization for GNB and GPC cases was 56 and 44.4 days respectively (*P*=0.3).

**Conclusion:**

The GNB antibiotic coverage should be designed more carefully in post-neurosurgical meningitis especially in patients with recent corticosteroid therapy and elevated ESR and procalcitonin.

The various methods used to neurosurgical operation may lead to central nervous system infection or postoperative meningitis. According to studies, the probability of meningitis due to surgery is 0.3% - 25%, which can be the main reason for an increased time of hospitalization and treatment costs (1). Post-neurosurgical meningitis (PNM) is divided into two groups: post-neurosurgical bacterial meningitis (PNBM) and post-neurosurgical aseptic meningitis (PNAM) (2). Elderly patients are more vulnerable to meningitis caused by bacterial agents and have a lower prognosis. Some risk factors for bacterial meningitis after surgeries are patient’s age, underlying medical conditions, and patient’s immune system, duration of hospitalization and surgery (3). Although gram-positive bacteria, such as *Staphylococcus aureus* and *Coagulase negative staphylococcus* are the main causative agents of meningitis, today a wide range of gram-negative bacteria, especially Multi Drug Resistance (MDR) *Acinetobacter and klebsiella* species, also play an important role in developing this complication (4).

In order to be accurate and have an early diagnosis of causative agents of disease in cases of post-neurosurgical meningitis, and reduction of mortality rate due to this condition, we conducted a study of post-neurosurgical bacterial meningitis and investigate the effect of gram-positive and gram-negative bacteria on patients' clinical characteristics, laboratory indexes, clinical measures, mortality rate and prognosis.

## Methods

In this study, among the 2667 neurosurgical patients who underwent craniotomy or endoscopy at Imam Khomeini Hospital complex we included 45 patients aged 12 to 72 years with positive CSF culture. CSF and blood samples were obtained from the patients with clinical manifestations including fever, headache, neck stiffness and nervous symptoms in sterile condition and transferred to a hospital laboratory for bacterial culture and biochemical analysis immediately. CSF samples were incubated for 5 days in BacT/ALERT 3D (bioMérieux, Marcyl’Etoile, France). Positive CSF samples then were sub cultured on 5% sheep blood agar, Eosin methylene blue (EMB) agar and chocolate agar and incubated in 37°C with 5% CO2 for 24 hours. Patients were excluded if yeast was isolated from their CSF culture. Identification and MIC method for antimicrobial susceptibility testing were performed using VITEK 2 system (bioMérieux, Marcy-l’Étoile, France). 43 patients received Cefazolin (95.6%) and 2 (4.4%) patients obtained ampicillin-sulbactam as preoperative prophylactic antibiotic. Biochemical analysis including leukocyte count, CSF protein concentration, serum C-reactive protein (CRP), erythrocyte sedimentation rate (ESR), serum procalcitonin level, CSF glucose, neutrophil, CSF lymphocyte and LDH levels, were evaluated. Other patient’s data including age, sex, duration and number of surgeries, GCS and type of surgery (elective or emergency), CSF leakage, history of corticosteroid therapy and their past medical history were recorded according to patient’s chart. This study was approved by Tehran University of Medical Sciences Ethics Committee (IR.TUMS.IKHC.REC.1396.2738). 

Data were analyzed by SPSS 18.0 software. Descriptive statistics was used to determine the incidence of meningitis based on different groups and multiple logistic regression test was used to determine the relationship between the type of meningitis and other indices like the success rate of treatment.

## Results

From April 2014 to May 2017, a total of 2077 and 590 patients were admitted to Imam Khomeini Hospital Complex for craniotomy and endonasal endoscopy surgeries respectively. Of these, 45 patients were enrolled in the study with positive cerebrospinal fluid (CSF) culture. The mean age of patients was 40.9 years with a minimum of 12 years and a maximum of 72 years. According to this study, 11.1% of patients had a history of corticosteroid therapy and 11.1% underwent emergency surgery. 35.6% of patients experienced cerebrospinal fluid leakage after surgery and 56.8% of them had extra ventricular or lumbar drainage. 6.7% of cases had surgery site infection after surgery. 28 (62.2%) were males and 17 (37.8%) were females. 17 (37.8%) of studied patients had a history of previous surgery. Fever was the most prevalent symptoms. Headache and loss of consciousness were the second most common symptoms among the patients. 38 (84.4%) patients had normal GCS (>15) while (15.6%) other cases had altered GCS (<15). Demographic and clinical characteristics of these 45 patients are shown in table 1. Pituitary adenoma (n=6 13.3%), tumor recurrence (n=5, 11.1%), chordoma (n=4, 8.9%), were the most common underlying diseases among the patients, respectively. CSF smear was positive in 4 cases including gram-positive cocci. Cerebrospinal fluid culture was positive in 45 patients. Of the implicated pathogens, gram negative bacteria (55.5%) (N=25) were the most common organisms causing meningitis followed by gram positive bacteria (42.2%) (n=19). One patient suffered from fungal meningitis caused by *Candida albicans* (n=1, 2.2%). The predominant gram negative pathogens were *klebsiella pneumoniae* and *Acinetobacter baumanni* .Also, the most common gram positive pathogen were *Coagulase Negative Staphylococci* (CoNs), *Staphylococcus aureus*. Table 1 shows the distribution of implicated pathogens. In vitro antibiotic sensitivity pattern of gram negative isolates showed 14% of *Klebsiella* strains produced carbapenemase and all *Acinetobacter* isolates were resistant to imipenem. *Coagulase Negative Staphylococci* and *Staphylococcus aureus* were the most prevalence gram positive isolates. 33.4% of *staphylococcus aureus* and 25% of *Coagulase negative staphylococcus* showed resistance to Vancomycin .87.5% 0f CoNs were resistant to linezolid whereas all Staphylococcus aureus strains were sensitive toward this antibiotic. Demographic data of PNBM caused by gram negative and gram positive bacteria are presented in table 2. No significant difference was observed between patient’s age, gender, surgical type, GCS and other mentioned data in table 4 and post-operative meningitis except corticosteroid therapy (P=0.03).

**Table 1:** Distributions of pathogens

**Isolated pathogens**	**Frequency**	**Percentage**
**Gram negative bacilli**		
*Klebsiella pneumoniae*	14	31.1
*Acinetobacter baumanni*	4	8.9
*Pseudomonas aeruginosa*	2	4.4
*Escherichia coli*	2	4.4
*Klebsiella oxytoca*	1	2.2
*Enterobacter cloacae*	1	2.2
*Enterobacter aerogenes*	1	2.2
**Gram positive **		
*Coagulase Negative Staphylococci*	8	17.8
*Staphylococcus aureus*	6	13.3
*Streptococcus pneumoniae*	1	2.2
*Non-hemolytic streptococcus*	1	2.2
*Staphylococcus haemolyticus*	1	2.2
*Bacillus *species	1	2.2
*Candida albicans*	1	4.4
Total	45	100

**Table 2:** Comparing of demographic/clinical data between GNB and GPC groups

		Gram negative bacteria n (%)	Gram positive bacteria n (%)	P-value
**Gender**	male	18 (64.3%)	10 (35.7%)	0.1
	female	7 (43.8%)	9 (56.3%)	
**Craniotomy**		19(76%)	14(70%)	0.33
**Endoscopy**		6(24%)	4(20%)	
**Surgical type**	Urgent	3(12%)	3(15.8%)	0.13
	Non urgent	22(88%)	16(84.2%)	
**Leakage**	Yes	11(44%)	4(21.1%)	0.11
	No	14(56%)	15(78.9%)	
**Superficial Surgical Site Infection**	Yes	3(12%)	-	0.28
	No	22(88%)	19(100%)	
**Corticosteroid use**	Yes	5(20%)	-	0.03
	No	20(80%)	19(100%)	
**Drainage**	external ventricular drain	11(44%)	5(26.3%)	0.06
	lumbar drain	4(16%)		
	Both	2(8%)	1(5.3%)	
	None	8(32%)	13(68.4%)	
**Clean**	Clean	18(72%)	15(78.9%)	0.7
	Contaminated	7(28%)	4(21.1%)	
**Re-surgery**	Yes	20(80%)	8(42.1%)	0.9
	No	5(20%)	11(57.9%)	
**Age**	Mean±SD	40.69±15.89	41.56±15.96	0.86
	Med(min-max)	40.00(12-70)	37.00(18-72)	
**GCS**	Mean±S.D	13.54±3.07	14.67±1.41	0.11
	Med(min-max)	15(6-15)	15(9-15)	
**Outcome**	Death	6(25%)	2(10.5%)	0.2
	Discharge	18(75%)	17(89.5%)	

Serum inflammatory parameters, erythrocyte sedimentation rate (ESR) and procalcitonin (PCT), were significantly higher in GNB cases compared GPC cases (P=0.01, P=0.02, respectively). Other inflammatory markers of serum including C reactive-protein (CRP), White blood Cells and also serum glucose level did not show significant differences (table 3). CSF analysis of GNB and GPC patients is shown in table 4. 

Despite the significant differences observed in patients’ serum biomarkers, no remarkable difference was observed in CSF protein levels (P=0.6) nor in CSF WBC levels (P=0.3).

**Table 3:** Comparing of laboratory data between GNB and GPC groups

	Gram negative (Mean±SD)	Gram positive (Mean±SD)	T-value(p-value)
**ESR** ** (** **mm/h)**	86.8±29	59.5±31.8	2.6(0.01)
**CRP** ** (** **mg/L)**	80.2±40.7	66.4±32.9	1(0.2)
**WBC(mm3)**	15707±4465	15500±4714	0.1(0.8)
**Glucose** ** (** **ng/dl)**	48.5±27.3	50.2±32.8	0.18(0.8)
**Procalcitonin (ng/ml)**	13.1±28.1	0.8±2	2.2(0.02)

**Table 4:** Comparing of CSF characteristics between GNB and GPC groups

Z-value (p-value)	Gram positive	Gram negative		
**0.4 (0.6)**	117±100	494±2004	Protein(ng/dl) (Mean±SD)	
**0.9 (0.3)**	861±1178	5894±15968	WBC(mm3) (Mean±SD)	
**0.4 (0.4)**	351±287	778±2127	LDH(mmol/L) (Mean±SD)	
**0.435**	70.92±29.29	77.00±17.05		Neutrophil Mean±S.D Med (min-max)
	86.0 (0-100)	80.50 (40-100)		
**0.752**	21.08±1.04	23.00±17.05		Lymphocytes Mean±S.D

The duration of hospitalization in GNB patients was longer compared to GPC patients, so that the first group was hospitalized for averaged 56 days and the second group for averaged 44.4 days (P=0.3).

## Discussion

Postoperative meningitis with an incidence rate of 0.3% to 25% is one of the most challenging hospital-acquired infection complications all over the world which is associated with high mortality, and requires early diagnosis and appropriate antibiotic therapy (1). Traditional methods (bacterial culture and gram staining) are the gold standard for the diagnosis of meningitis; however, bacterial culture is time consuming and also may be false negative (5). As B. K. Das et al. reported that 5 *Escherichia. coli* and 5 *Streptococcus pneumonia* isolates were identified only by latex agglutination method and not by culture (2, 6). The severity of infections caused by gram-negative bacteria seems to be higher than that of gram-positive bacterial meningitis. As seen in this study, almost all laboratory indexes of GNB patients were higher than GPC patients. In general, the length of the hospital stay in GNB patients was longer and the treatment of patients was much more difficult than that of GPC cases due to several protective layers in their structure and their high ability to acquire antibiotic resistance genes. We attempt to present a prompt way to detect the etiology agent of meningitis by comparing laboratory indexes in patients with post-operative meningitis caused by gram-positive and gram negative bacteria. Several studies aimed to investigate laboratory indexes for correct and early diagnosis of meningitis after surgery have been done. 

Serum level of PCT in GNB cases was higher than that in patients in the second group. Similar to our findings, X Y Wang et al. by studying of 813 cases of CNS infection after craniocerebral surgery reported that serum PCT and CRP level in gram negative infected patients were higher than that of gram-positive patients. While, CSF glucose concentration in gram positive group was higher than gram negative group (7). Wen Li, et al., in a study of 135 patients with meningitis, concluded that CSF PCT level was higher in patients with gram-negative bacterial meningitis than in those with gram-positive bacteria although there was no significant difference. (8). Hong-Yuan Shen et al. stated that serum PCT concentration has more diagnostic and clinical value than in CSF (9). 

It is stated that serum CRP level is a better indicator for predicting bacterial meningitis because the level of this protein in CSF indicates only the degree of impairment of the blood-brain barrier (10). In our study, the increased level of serum CRP in GNB cases albeit non-significant was observed while ESR level was significantly elevated in GNB patients compared to GPC group which can be helpful in predicting causative agent. Studies have shown that CSF CRP level is significantly higher in gram-negative bacterial meningitis than in gram-positive meningitis. However, the level of this biomarker in the serum of patients in these two groups was not significant. (11, 12).

 Similar to our results, previous studies reported that the improvement rate of gram-positive infected patients was higher than those who were infected with gram negative (13). Other laboratory factors like CSF protein and serum creatinine and urea can be useful in predicting causative meningitis agents. As A. Neuberger concluded that in those patients who died or developed neurological dysfunction caused by gram-negative meningitis, CSF protein and serum glucose, blood urea nitrogen and blood creatinine levels were higher than healthy subjects, while CSF glucose level is lower. We observed that CSF protein level in patients with gram negative CNS infection was higher than that of other group but this difference was not significant. More studies have shown that gram positive infected patients have increased the level of consciousness than gram-negative cases. These results are consistent with our findings that GCS level of GNB patients is lower compared to GPC patients (14). 

Erman et al. presented that the steroid use before surgery was not significantly associated with the progression of surgical site infection in patients undergoing neurosurgery (15). While it is reported by Irene S. Kourbet et al. that preoperative steroid use can be a risk factor for post-neurosurgical meningitis. It is also mentioned that ventricular drain and CSF leak have significant correlation with post-operative meningitis (1). As observed in our study preoperative steroid therapy has significantly effect on the development of post-neurosurgical meningitis specially with gram negative bacilli but no remarkable correlation was found between meningitis and CSF leak and the presence of drain in two groups. The incidence of meningitis after neurosurgery caused by multi-drugs resistant (MDR) gram-negative bacilli is concerning. Among these pathogens carbapenem resistance *Acintobacter baumanni* and *Klebsiella pneumonia* have a significant role in mortality. According to our study all *A.baumannii* isolates and 14% of *Klebsiella pneumonia* were Carbapenemase Producing. 

Yi-jun Shi et al. reported that among the patients with *Enterobacteriaceae* meningitis, *Klebsiella pneumoniae,* followed by *E.coli* were the most common microorganisms isolated from the cerebrospinal fluid of patients. 37.8% of isolates were Extended Spectrum Beta-Lactamase (ESBL) producing. 80% of all isolates were sensitive to imipenem and meropenem (16). 

It is reported by M. S. Raza et al. that 58.3% of *A.baumannii* and 60% of *K.pneuomoniae* isolates were susceptible to Colistin and 41.6% of *A.baumannii* and 60% *of K.pneumoniae* were susceptible to Imipenem and meropenem (17). Consistent to our study *Acinetobacter* species, *Pseudomonas aeruginosa* and *Klebsiella pneuomoniae* were the most common gram negative pathogens (18). 

Reports indicate that in addition to serum and cerebrospinal fluid biomarkers, cytokines and chemokines can also be used to predict the etiology of meningitis. Previous study demonstrated that the children with gram positive CSF shunt infection have higher levels of interleukin (IL) 10, vascular endothelial growth factor and interleukin-17A which leads to neutrophil predominance (19). Overall, in patients with nosocomial meningitis IL-8, IL-12 and lactate levels were higher in CSF of postoperative patient and the levels of IL-13 and IFN gamma are lower than normal (20).

In conclusion we suggest that GNB antibiotic coverage should be designed more carefully by clinicians in patients with post-neurosurgical meningitis who have a history of corticosteroid therapy and their ESR and PCT serum levels are higher than normal.
